# Preclinical approaches in vulvovaginal candidiasis treatment with mucoadhesive thermoresponsive systems containing propolis

**DOI:** 10.1371/journal.pone.0243197

**Published:** 2020-12-11

**Authors:** Amanda Pohlmann Bonfim, Karina Mayumi Sakita, Daniella Renata Faria, Glaucia Sayuri Arita, Franciele Abigail Vilugron Rodrigues Vendramini, Isis Regina Grenier Capoci, Andressa Gimenes Braga, Rafaela Said dos Santos, Marcos Luciano Bruschi, Tania Cristina Alexandrino Becker, Admilton Gonçalves de Oliveira Junior, Érika Seki Kioshima, Patrícia de Souza Bonfim-Mendonça, Terezinha Inez Estivalet Svidzinski

**Affiliations:** 1 Medical Mycology Laboratory, Clinical Analyzes and Biomedicine Department, State University of Maringa, Maringa, PR, Brazil; 2 Department of Pharmacy, State University of Maringa, Maringa, PR, Brazil; 3 General Pathology Laboratory, Healthy Basic Sciences Department, State University of Maringa, Maringa, PR, Brazil; 4 Electron Microscopy and Microanalysis Laboratory, State University of Londrina, Londrina, PR, Brazil; University of Porto, PORTUGAL

## Abstract

Vulvovaginal candidiasis (VVC) is a common vaginitis that affects women, especially in childbearing age, caused by *Candida albicans* in almost 80% of cases. Considering the limited drug arsenal available and the increasing fungal resistance profile, the search for new therapeutic sources with low toxicity and easy administration should be supported. Propolis has been used as a traditional medicine for multiple diseases, considering its particular composition and pharmaceutical properties that permits its wide applicability; it has also emerged as a potential antifungal agent. Thus, this study performed an *in vitro* and *in vivo* investigation into the efficacy of a new mucoadhesive thermoresponsive platform for propolis delivery (MTS-PRPe) in a preclinical murine model of VVC treatment caused by *C*. *albicans*. The methodologies involved chemical analysis, an assessment of the rheological and mucoadhesive properties of propolis formulations, *in vitro* and *in vivo* antifungal evaluations, histological evaluations and electron microscopy of the vaginal mucosa. The results demonstrated the antifungal activity of propolis extract and MTS-PRP against the standard strain and a fluconazole-resistant clinical isolate of *C*. *albicans*, in both *in vitro* and *in vivo* assays. These results were similar and even better, depending on the propolis concentration, when compared to nystatin. Thus, the formulation containing propolis exhibited good performance against *C*. *albican*s in a vulvovaginal candidiasis experimental model, representing a promising opportunity for the treatment of this infection.

## Introduction

Vulvovaginal candidiasis (VVC) is an opportunistic fungal infection caused by *Candida* species, mainly *C*. *albicans* [1]. After bacterial vaginosis, VVC is the second most common cause of vaginal infection [2]. According to reports, at least 75% of women of childbearing age, present VVC once in a lifetime, among which 9% of them will develop into recurrent VVC [3]. *C*. *albicans* is undoubtedly the most frequent fungal pathogen species responsible for superficial and invasive human infections at different anatomical sites, reported in studies worldwide. It is known as a strongly pathogenic yeast with virulence factors such as the capacity to adhere to epithelia and mucous membranes, dimorphism, thermotolerance, and the production of proteases and phospholipases [4]. Thus, if the microflora is unbalanced or immunological defenses are compromised, *C*. *albicans* can be pathogenic [5]. The clinical expression of this infection includes typical leukorrhea, vulva and vaginal burning, itching, dysuria, dyspareunia, frequent micturition, and others [6]. In this context, VVC is recognized as a serious challenge to public health with a heavy socio-economic and medical impact [5]. The most common drugs for the clinical treatment of VVC are nystatin (NYS), fluconazole (FCZ), and miconazole, but the complexity of VVC physiopathology often leads to a gradual increase in drug resistance during the course of treatment [7, 8]. Besides resistance, other frequently used drugs such as azole derivatives, polyenes, and echinocandins are responsible for undesirable side effects and toxicity [9–11].

Propolis (PRP) is a complex compound produced by honeybees (*Apis mellifera*) from plant exudates; it consists of balsamic gums and resinous materials. Its chemical composition includes multiple substances such as flavonoids, terpenoids, phenylpropanoids, and many others [12]. Reports suggest that phenolic compounds are the main components responsible for the antimicrobial activity of propolis extracts [13]. Improved by pharmaceutical technology, many different extraction processes have been reported for propolis extract (PRPe) [14]. Therefore, the characterization of the chemical and antifungal properties of new types of propolis extract is important to pharmacists who work on the development of new drugs. In this context, mucoadhesive drug delivery systems have been shown to be attractive and flexible options because they permit drug administration via multiple routes (ocular, nasal, vaginal, rectal, and others) at various concentrations to improve availability. Some of the advantages of using mucoadhesive drug delivery systems includes improved drug bioavailability, reduced administration frequency, increased residence time, simplified administration, and the possibility of targeting particular tissues and body sites [15].

As the number of commercial antifungal agents is limited, and coupled with the increasing rates of *C*. *albicans* infections, it is important to optimize studies focused on the development of new drugs to treat this infection, especially recurrent cases, with high effectiveness, low cost, and minimum adverse effects. Some semi-solid mucoadhesive formulations have been proposed for vaginal propolis delivery [16]. These systems are composed of only one or a mixture of mucoadhesive polymers such as acrylic-acid derivatives, chitosan, cellulose derivatives, or polysaccharides [15, 16]. These systems have been characterized regarding their antimicrobial activities, but their mechanical, textural, and rheological properties have not been investigated properly. This could impair system performance during administration and maintenance at the site, as well as the drug delivery profile. Therefore, we designed binary polymeric systems composed by poloxamer 407 and Carbopol 934P that display mucoadhesive and thermoresponsive properties and have been shown to be a promising platform for the vaginal delivery of propolis [17]. In this study, we assessed for the first time the efficacy of mucoadhesive thermoresponsive systems containing propolis (MTS-PRPe) for the treatment of VVC caused by *C*. *albicans*.

## Materials and methods

### Preparation and characterization of mucoadhesive thermoresponsive systems

The systems were prepared using poloxamer 407 (P407) (20%, w/w) (Sigma, St. Louis, USA) and Carbopol 934P (C934P) (0.15%, w/w) (BF Goodrich, Ohio, USA). The carbomer was dispersed in purified water, with mechanical stirring. After total dispersion of C934P, the polymer P407 was added and the preparation was stored at 4°C for 12 h to ensure complete hydration. Afterwards, the preparation was stirred, ensuring the complete mixing of the polymers, and the pH was set to 7.0 using triethanolamine (Galena, Campinas, SP, Brazil). Propolis extract (PRPe) was prepared as described in S1 Data and added to the system at a concentration of 14 or 16% (w/w) with mechanical agitation. All formulations were stored in amber jars at 4°C for at least 24 h before further analysis [14].

The formulations were analyzed regarding pH and relative density [18]. The preliminary sol-gel transition temperature (preliminary Tsol/gel) was determined according to Choi and collaborators [19].

### Rheological analysis

The rheological analysis of formulations was accomplished using a controlled stress rheometer (MARS II, Haake Thermo Fisher Scientific Inc., Newington, Germany) with parallel steel cone-plate geometry (60 mm, cone code L09006 C60/1°TiL, separated by a gap of 0.052 mm).

#### Continuous shear (flow) rheometry

Flow analysis of formulations was performed at 5°C, 25°C, and 37°C ± 0.1°C. The thermometer was set in flow mode, then the sample was carefully placed on the lower plate and allowed to equilibrate for at least 1 min prior to analysis. Flow curves were measured over shear rates ranging from 0 to 2000 s-1 (increased over a period of 150 s, held at the upper limit for 10 s, and then decreased over a period of 150 s). In each case, the continuous shear properties of at least five replicates were determined, and the up curves were modeled using the Oswald-de-Waele equation (power law) described in Eq 1 [14, 20]:

$$\tau =k.\gamma^{n}$$ Eq 1

where τ is the shear stress (Pa), k is the consistency index [(Pa.s)n], γ is the shear rate (s-^1^) and n is the flow behavior index (dimensionless).

The yield value of each formulation was evaluated by the Casson (Eq 2) and Herschel-Buckley (Eq 3) rheological models [19].

$$\tau =\sqrt[{M}]{(\tau_{0+(\gamma .n_{p}))n}^{n}}$$ Eq 2

where τ is the shear stress (Pa), n is the flow behavior index (dimensionless), τ0 is the yield stress (Pa), γ is the shear rate (s-^1^) and ηp is the Casson plastic viscosity.

$$\tau =\tau_{0+k.\gamma^{n}}$$ Eq 3

where τ is the shear stress (Pa), τ0 is the yield stress (Pa), k is the consistency index [(Pas)n], γ is the shear rate (s-^1^) and n is the flow behavior index (dimensionless). The hysteresis area of each system was also calculated using Rheo Win 4.10.0000 (Haakes) software.

#### Oscillatory rheometry

The oscillatory rheometry of all formulations was performed at 5°C, 25°C and 37°C ± 0.1°C. A sample of each formulation was carefully applied on the plate as previously described. After determination of the linear viscoelastic region of each formulation, frequency sweep analysis was evaluated from 0.1 to 10.0 Hz. The storage modulus (G’), loss modulus (G″), dynamic viscosity (η’), and the loss tangent (tan δ) were calculated using Rheo Win 4.10.0000 (Haake) software. In each case, the dynamic rheological properties of at least three replicates were evaluated [26].

In addition, the sol-gel transition temperature (Tsol/gel) of the formulations was also performed in oscillatory mode using temperature ramp analysis. After the determination of the linear viscoelastic region of each formulation at 5°C and 60°C, a temperature sweep analysis was performed over the temperature range of 5–60°C at a defined frequency (1.0 Hz) and rate of heating 10°C/min using a controlled stress. G’, G”, viscosity η', and tan δ were calculated using Rheo Win 4.10.0000 (Haake) software. Tsol/gel was determined for all formulations in which the η’ increased with increasing temperature. Moreover, the temperature at which G′ was halfway between the values for the solution and the gel was considered Tsol/gel [14, 15].

### Strains and culture methods

The *in vitro* susceptibility tests were performed with PRPe and conventional antifungals against *C*. *albicans* ATCC 90028 from the American Type Culture Collection (ATCC) and 88 *C*. *albicans* clinical isolates from patients with VVC that belong to the archive collection of the Laboratory of Medical Mycology, Universidade Estadual de Maringa, Parana, Brazil. These isolates were divided into three groups according to clinical manifestations: 40 clinical isolates from the colonized group (asymptomatic), 27 from the VVC group (single symptomatic episode of VVC infection), and 21 from the recurrent VVC group (RVVC) that means more than three episodes of VVC infection in the last year. Prior to testing, the strains were subcultured in Sabouraud dextrose agar (SDA; Becton, Dickinson and Company, Sparks, MD) and incubated at 37°C for 24 h.

The inoculum preparation was performed through the suspension of the yeast in phosphate-buffered saline (PBS; 0.1 M; pH 7.4), adjusting the concentration by counting in a Neubauer chamber to 2.5–5.0 x 106 CFU mL-1. Further dilutions were prepared until the desired final inoculum value was reached (2.5–5.0 x 103 CFU mL-1) [20, 21].

### Antifungal agents

The following antifungals and compounds were used in the *in vitro* susceptibility tests: nystatin (NYS; Sigma, St. Louis, MO, USA), fluconazole (FCZ; Pfizer, Brazil), PRPe, and the MTS-PRPe systems at 14% (F14%) and 16% (F16%).

### Antifungal susceptibility assays

The minimum inhibitory concentration (MIC) of FCZ and NYS and the minimum fungicidal concentration (MFC) of PRPe were determined by the broth microdilution method for 88 clinical isolates and one ATCC strain, and the MFC of F14% and F16% were assessed by the broth macrodilution method for four clinical isolates. All assays were performed according to Clinical and Laboratory Standards Institute (CLSI) document M27-A3 [22], adapted for natural compounds [17]. The final concentration of both FCZ and NYS ranged from 0.125 to 64 μg/mL, PRPe 5.23 to 2680 μg/mL of total polyphenol content (TPC) expressed as gallic acid equivalents, MTF-PRPe at 14% (F14%) ranged from 138.57 to 2217.18 μg/mL of TPC expressed as gallic acid equivalents, and MTF-PRPe at 16% (F16%) from 158.40 to 2534.40 of TPC expressed as gallic acid equivalents.

According to CLSI supplement M27-A3, the MIC was defined as the lowest concentration of the antifungal agent that was able to inhibit 50% of fungal growth for FCZ and 90% for NYS, compared to the positive control without drugs [22]. The cut-off levels of susceptibility to FCZ and NYS were utilized according to CLSI supplement M27-S4 [23] and Dalben-Dota et al. [24] to identify strains as susceptible (S), dose-dependent susceptible (DDS), and resistant (R) to FCZ (S ≤ 2 *μ*g/mL, DDS = 4 *μ*g/mL, R ≥ 8 *μ*g/mL) or NYS (S ≤ 4 *μ*g/mL, DDS = 8–32 *μ*g/mL, R ≥ 64 *μ*g/mL).

The antifungal activity of PRPe, F14%, and F16% were evaluated according to the MFC. For this, aliquots from each well of the PRPe microdilution assay and the F14% and F16% macrodilution assay were withdrawn and seeded in Petri dished with SDA medium to determine the MFC and the quantitative reduction in colony forming units (CFU/mL). The MFC was defined as the lowest concentration of the antifungal agent solution that inhibited 100% yeast growth.

### Murine candidiasis vaginal model

The experiments were carried out according to the institutional regulations and were approved by the Institutional Ethics Committee for animal experimentation of the Universidade Estadual de Maringa, Brazil (approval number CEUA 4717180616, from 08/05/2016). The animals were treated according to the Guide for the Care and Use of Laboratory Animals from the Institute of Laboratory Animal Resources/USA [25]. Female Balb/c mice (6–8 weeks of age and weighing 20–22 g) were obtained from Bioterio Central of Universidade Estadual de Maringa and housed under standard conditions.

First, pseudoestrus status was achieved in the animals, for optimal *Candida* colonization. For this, 0.1 mg of estradiol valerate (17 β-estradiol valerate) previously dissolved in sesame oil (Sigma-Aldrich) was injected subcutaneously in the dorsal area of each animal, followed by a massage to facilitate the dispersion of the suspension. This procedure was performed 72 h before infection and repeated once a week during the treatment period, to guarantee estrogenic vaginal conditions.

For the VVC infection, 30 μL of a suspension in PBS containing a standard inoculum of 1.0x106 CFU of *C*. *albicans* (ATCC 90028) or a clinical isolate resistant to FCZ from a woman with RVVC were inoculated by inserting the pipette tip about 5 mm deep into the vaginal lumen [26].

### Treatment protocol

#### Antifungal agents

The following antifungal and compounds were used for *in vivo* treatment assays: PRPe, MTS without propolis (C2), F14%, F16% and nystatin cream (100,000 IU/4 g; Neoquimica/Brainfarma, Goias, Brazil).

#### Treatment groups

The infected animals were divided into seven groups (n = 8): C1 (control group, i.e. infected, untreated animals), C2 (infected animals treated with MTS without propolis, twice a day), PRPe (infected animals treated with PRPe, twice a day), F14% (infected animals treated with MTS-PRPe at 14%, twice a day), F16% (infected animals treated with MTS-PRPe at 16%, twice a day), F16%-1x (infected animals treated with MTS-PRPe at 16%, once a day), and NYS (infected animals treated with nystatin commercial cream, twice a day).

#### Treatment protocol

Each group was treated twice a day, with a 12 hour interval (except for the F16%-1x group, which was treated once a day and the control group) with 30 μL (0.123 μg of TPC expressed as gallic acid equivalents) for PRPe and 60 μL for F14% and F16% (0.266 and 0.304 μg of TPC expressed as gallic acid equivalents, respectively), for 7 and 14 days. In parallel, 16 non-infected animals were split into two groups of 8 animals each and treated with the two formulations (F14% and F16%) for 7 and 14 days to perform an *in vivo* toxicity evaluation.

After 7 and 14 days of treatment, the animals of each subgroup (n = 8) were euthanized (isoflurane overdose inhalation), the dissected vaginal tissue was evaluated for fungal burden (CFU) and a histological analysis in half of the animals, while the other half was evaluated by fungal burden (CFU) and scanning electron microscopy (SEM).

#### Fungal burden quantification

After establishing experimental vaginal candidiasis in mice and finishing the treatment protocol, the fungal burden quantification was performed. The vaginal tissues were aseptically removed, weighed and mechanically homogenized in sterile lysis buffer. Serial 10-fold dilutions of the homogenates in PBS were placed on SDA and incubated for 48 h at 35°C to evaluate the efficacy of the treatment by a reduction in the vaginal tissue fungal burden, presented as log10 CFU/g of tissue.

#### Histopathological studies

The vaginal tissue removed from each animal was fixed in 4% paraformaldehyde, paraffin embedded and cut into thin sections (5 μm). The sections were stained with Grocott-Gomori's methenamine silver (GMS) to visualize fungi, and counterstained with hematoxylin and eosin (H&E) for the characterization of host cells [27]. Additionally, to assess the toxicity of the studied formulations, vaginal tissue from non-infected animals was stained with H&E. All stained slides were immediately washed, preserved with mounting medium and sealed with a coverslip. Then, they were observed and photographed using a binocular light microscope (Motic BA310), coupled to a camera (Moticam 5) and to a computer, using Motic Images Plus 2.0 software. The analyzed parameters (epithelial changes and presence of *C*. *albicans*) were descriptive and examined by a single researcher oriented by a pathologist and both were blinded to the analysis of the groups.

#### Ultrastructural evaluation

Scanning electron microscopy (SEM) was performed following the protocol of de Oliveira et al. [28]. Briefly, for SEM preparation, samples collected at 24 h were fixed by immersion in 2.5% glutaraldehyde, then 2% paraformaldehyde in 0.1 M sodium cacodylate buffer, post-fixed in 1% OsO_4_, and dehydrated in an ethanol series (70, 80, 90, and 100%). Samples were critical-point dried with CO_2_ (BALTEC CPD 030 Critical Point Dryer), coated with colloidal gold (BALTEC SDC 050 Sputter Coater) and observed under a scanning electron microscope (FEI Quanta 200).

#### Cytotoxicity evaluation of mucoadhesive thermoresponsive formulations containing propolis

Two groups of four non-infected animals each were treated with mucoadhesive thermoresponsive formulations containing propolis at 14% (F14%) and 16% (F16%) to cytotoxicity test. These animals were treated for 7 and 14 days, then euthanized following the same protocol described above. The vaginal fluid from each animal was collected, centrifuged, and stored at -80°C. The vaginal fluid from each animal was collected, centrifuged, and stored at -80°C. A CBA Mouse Th1/Th2/Th17 Cytokine kit (BD™ Biosciences Mountain View, CA, USA) was used according to the manufacturer’s instructions. Samples were tested for the following cytokines: interleukin-2 (IL-2), interleukin-4 (IL4), interleukin-6 (IL-6), interferon-γ (IFN-γ), tumor necrosis factor (TNF), interleukin-17A (IL-17A), and interleukin-10 (IL-10), and analyzed using a FACS Calibur flow cytometer and Cell Quest software (BD Biosciences, San Jose, CA, USA). In this phase, we tested cytotoxicity of MTS formulations in animal groups without infection. The results were analyzed according to a standard cytokine curve using the software FCAP Array 3.0, and the results are expressed as mean ± standard deviation. Only the detected cytokines and statistically different results are represented in the graphs.

### Statistical analyses

Student’s t-test was used for the comparison between the treated and untreated group and one-way analysis of variance (ANOVA) was used for multiple comparisons, applying the Bonferroni multiple-comparisons test. The data were analyzed using Prism 6.0 software (GraphPad, San Diego, CA, USA). Values of p<0.05 were considered statistically significant. All tests were performed at least in duplicate.

## Results

### Mucoadhesive thermoresponsive system formulations: Physicochemical characterization

According to Table 1, the combination of thermoresponsive and mucoadhesive polymers P407 and C934P, respectively, showed good physicochemical characteristics.

**Table 1:** Physicochemical analysis performed for formulations containing different concentrations of PRP extract.

	Analyzes (Mean ± s)			
Formulation (PRP extract content %, w/w)	Preliminary gelation temperature (°C)	Relative density (g/mL)	pH	Theoretical total polyphenols content (%, w/w)
F14 (14%)	17.33 ± 0.5774	1.0182 ± 0.0026	7.10 ± 0.1266	0.3757 ± 0.0140
F16 (16%)	15.67 ± 0.5774	1.0190 ± 0.0002	7.18 ± 0.1054	0.4293 ± 0.0161
FB (no extract)	19.33 ± 0.5774	1.0301 ± 0.0016	7.00 ± 0.0000	

FB: mucoadhesive thermoresponsive system without propolis; F14: mucoadhesive thermoresponsive system containing propolis at 14%; F16: mucoadhesive thermoresponsive system containing propolis at 16%.

The systems had their pH set to 7.0 using triethanolamine due to the presence of Carbopol 934P, a polymer with acidic characteristics. This pH value utilized for the best mechanical and rheological characteristics of the systems [14, 17, 21]. Considering that the mouse vagina has a neutral pH, while the environment of the human vagina id acidic, as well as the small amount of formulation administered, the formulation pH cannot change this physiological condition due the gradual swelling of Carbopol chains. Moreover, the neutral pH of the formulations (14% and 16%) may balance the vaginal pH, reducing the marked acidity of VVC.

When the T_sol/gel_ of a vaginal formulation is lower than 20–25°C, a gel can be formed at room temperature, hindering handling and administration. On the other hand, if the T_sol/gel_ is higher than 37°C, the liquid can remain inside the vaginal cavity that can lead to flow and loss of the formulation from the site of action (Pereira et al., 2013). In this study, the systems displayed a preliminary sol-gel transition temperature near 20°C, indicating that they can be maintained under refrigerated temperatures, like many other medicines. However, the time spent preparing the formulation in the vaginal applicator was enough to increase the temperature, thereby reducing the risk of discomfort. Afterwards, the systems were applied before the sol-gel transition, ensuring a good distribution and contact with the vaginal mucosa.

**Fig 1:**
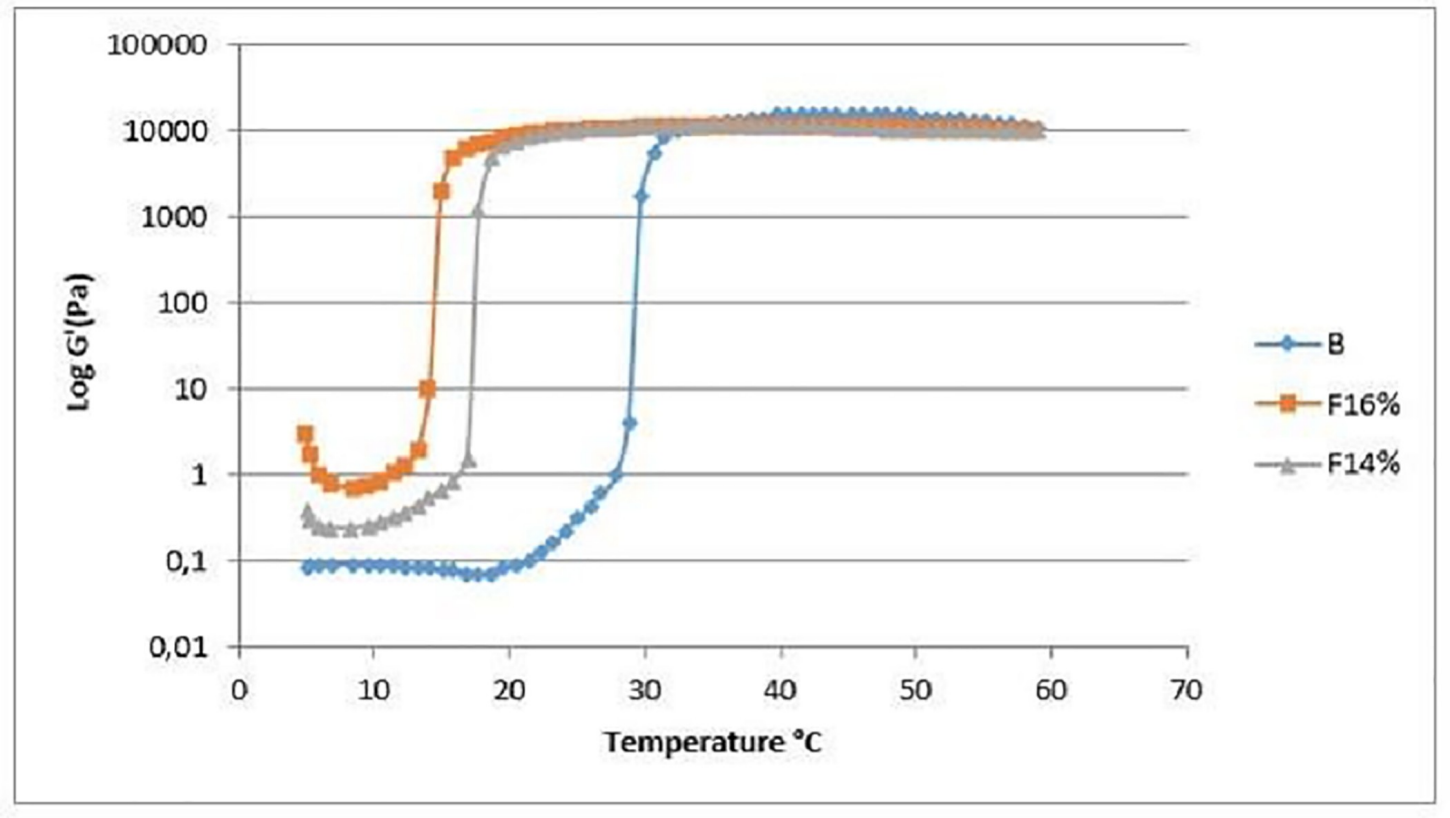
Gelling temperature (°C) as a function of the elastic modulus (G') of the formulations containing different concentrations of propolis extract (PRPe). Each curve represents the average of at least three replicates. The standard deviation bars were omitted to clarify the graph, but all showed a coefficient of variation lower than 15%. B: mucoadhesive thermoresponsive system without propolis; F14%: mucoadhesive thermoresponsive system containing propolis at 14%; F16%: mucoadhesive thermoresponsive system containing propolis at 16%.

For the T_sol/gel_, as shown in Fig 1, there was a decrease in temperature as the concentration of the extract increased in the mucoadhesive thermoresponsive system. Such mixtures are stored at low temperatures as aqueous dispersions, and when administered at body temperature the viscosity increases until reaching a plateau. These are stable systems and no phase separation was observed.

The polymer systems were characterized by rheology (oscillatory and continuous flow shear) to analyze their behavior at storage temperature (5°C), room temperature (25°C), and body temperature (37°C).

In the continuous shear rheology, the system without PRPe (B) presented non-Newtonian flow behavior, as did the other formulations (F14% and F16%) containing PRPe. In addition, all systems displayed plastic characteristics. The formulations with different concentrations of PRPe, at 5°C, presented a yield value where an initial stress is required for the formulation to flow [29, 30]. Moreover, all formulations displayed thixotropy at all temperatures (Table 2), with a hysteresis area of 209,566.67 Pa/s and 305566.67 Pa/s for the formulations F14 and F16 at 37°C, respectively; this was dependent on the temperature and presence of propolis extract. As the temperature increased, the thixotropy of formulation F16 increased. An increase in the amount of propolis extract increased the thixotropy of the formulations.

**Table 2:** Results of the mean of the consistency index (k), the flow index (n) and the yield value (τ_0_).

Temperature (°C)	Formulation	K (Pa.s)	n	τ_0_ (Pa)
	FB	459.2	0.8291	2348
	F14	1156.3	0.7677	5064
5	F16	1646.3	0.7659	7136
	FB	23883	0.5202	174400
	F14	74326	0.3879	303133
25	F16	74073	0.3444	296600
	FB	125300	0.3101	380733
	F14	67056	0.3679	228966
37	F16	58340	0.3913	241866

FB: mucoadhesive thermoresponsive system without propolis; F14: mucoadhesive thermoresponsive system containing propolis at 14%; F16: mucoadhesive thermoresponsive system containing propolis at 16%.

According to Table 2, the consistency index (k) increased with an increase in the extract concentration in the systems. The flow behavior index (n) presented values below 1, which confirms that the formulations were non-Newtonian. All the systems presented yield values (τ_0_), therefore all were plastic, and the yield value increased with an increasing concentration of the extract in the formulation and with increasing temperature.

At 5°C, the loss modulus (G") was larger than the elastic modulus (G'), i.e. the formulation was elastic-viscous. At temperatures of 25°C and 37°C, the formulations maintained a pattern, where G' was greater than G", and the different concentrations of the extract did not interfere. The dynamic viscosity (η') of the formulations increased as the temperature increased. For example, the formulation at 37°C increased in viscosity, which prevented flow. In addition, η' decreased with increasing frequency. The more concentrated formulation also showed an increase in viscosity. With an increase in frequency, the loss tangent (tan δ) decreased at 5°C, confirming that with increasing frequency the loss modulus increased in relation to the elastic modulus.

The polymeric systems presented properties that were dependent on temperature and oscillatory frequency, as well as on the presence and concentration of the extract. An increase in frequency significantly decreased the viscosity η'. For the G' and G'' moduli, the formulations at 5°C had smaller moduli than at 25°C and 37°C. In addition, the G'' modulus increased with increasing frequency at 5°C, but the G'' modulus decreased at 25°C and 37°C.

In general, as the extract concentration in the formulation increased, the G' and G" moduli, dynamic viscosity η', and loss tangent (tan δ) also increased.

### *In vitro* antifungal activity of PRP extract and mucoadhesive thermoresponsive systems containing propolis on *C*. *albicans* isolated from vulvovaginal candidiasis

For antifungal susceptibility testing with commercial drugs, 88 clinical isolates from women infected with *C*. *albicans* where divided into three groups according to the clinical manifestation: colonized group, VVC group, and RVVC group. These results were interpreted from the value of the MIC and are displayed in Table 3. Most of the clinical isolates showed susceptibility to NYS (97.5% of the colonized group; 100% of the VVC group; 95% of the RVVC group) and FCZ (97.5% of the colonized group; 100% of the VVC group; 95% of the RVVC group). Otherwise, 5% of isolates showed resistance to FCZ in colonized group, as well as in the RVVC group.

**Table 3:** Minimal inhibitory concentration of antifungal drugs against *Candida albicans* clinical isolates of vulvovaginal candidiasis.

Antifungals	*C*. *albicans*																	
	Colonized (n = 40)						VVC (n = 27)						RVVC (n = 21)					
	S		DDS		R		S		DDS		R		S		DDS		R	
	n	(%)	n	(%)	n	(%)	n	(%)	n	(%)	n	(%)	n	(%)	N	(%)	n	(%)
**NYS**	**39**	**97.5**	**1**	**2.5**	**0**	**-**	**27**	**100**	**0**	**-**	**0**	**-**	**20**	**95**	**1**	**5**	**0**	**-**
**FCZ**	**39**	**97.5**	**0**	**-**	**1**	**2.5**	**27**	**100**	**0**	**-**	**0**	**-**	**20**	**95**	**0**	**-**	**1**	**5**

NYS: nystatin; FCZ: fluconazole; S (susceptible): isolates with MIC≤ 4 for NYS; ≤2 for FCZ. DDS (dose-dependent susceptibility): isolates with MIC between 8 and 32 mg/mL for NYS; 4mg/mL for FCZ. R (resistant): isolates with MIC ≥ 64 mg/mL for NYS; ≥ 8 mg/mL for FCZ. *C*. *albicans* reference ATCC 90028 NYS MIC = 0.25 mg/mL and FCZ MIC = 0.125 mg/mL.

**Fig 2:**
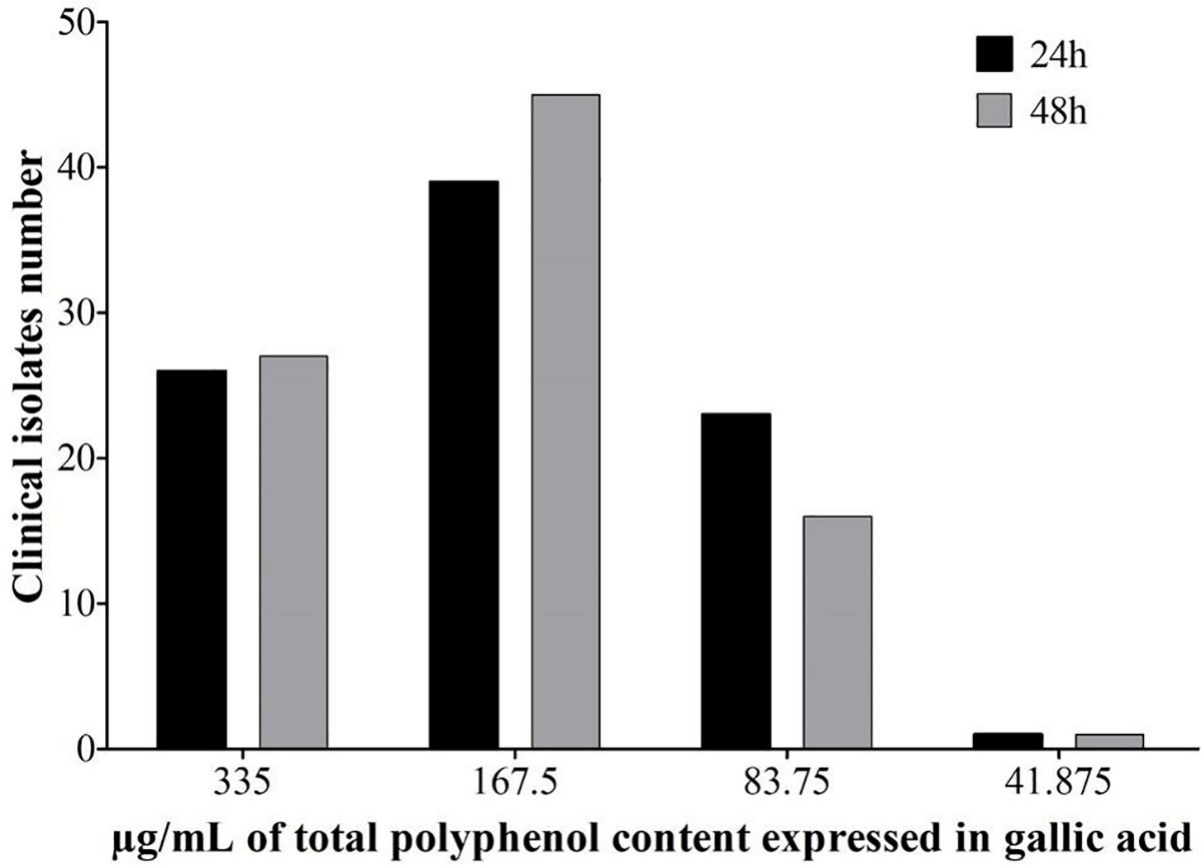
*In vitro* antifungal activity of propolis extract (PRPe) in clinical isolates of vulvovaginal candidiasis. Distribution of 88 *C*. *albicans isolates* and 1 reference strain ATCC 90028, according to minimum fungicidal concentration of PRPe in 24 hours and 48 hours. The data are expressed as number of clinical isolates inhibited at each concentration, of two independent experiments.

The MFC of PRPe for 88 clinical isolates and a standard strain (ATCC 90028) showed a similar response at two different reading times (24 and 48 h) (Fig 2). Most of the isolates (44–51%) were inhibited at the PRPe concentration 167.5 μg/mL, followed by 29–30% at 335 μg/mL, and 18–23% at 83.75 μg/mL.

**Fig 3:**
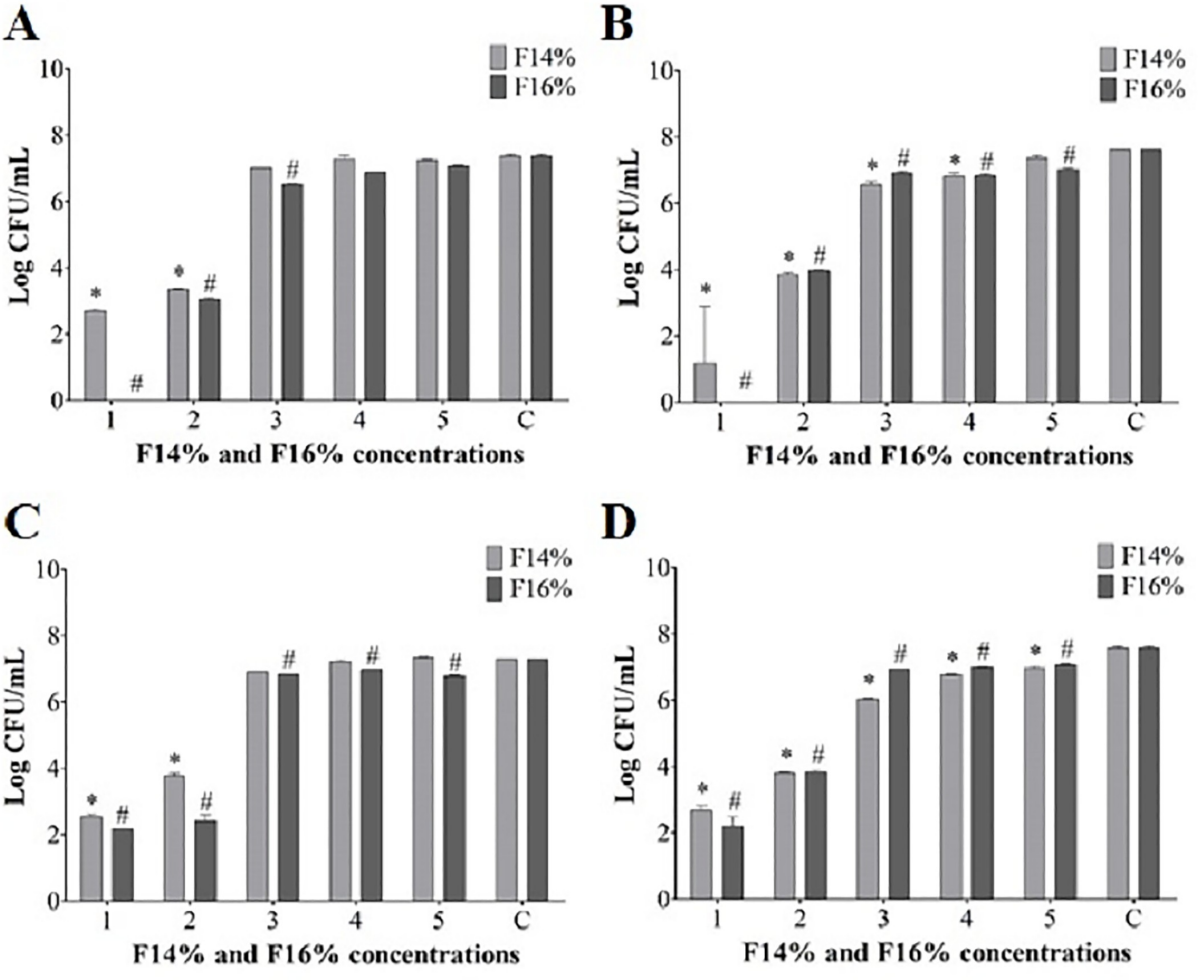
*In vitro* activity of mucoadhesive thermoresponsive formulations containing propolis at 14% (F14%) and 16% (F16%) in four *C*. *albicans* clinical isolates. **(**A,B,C,D) Quantitative logarithmic evaluation reduction of F14% and F16% at 24 hours. Control: positive control; inoculum without F14% or F16%. F14% 1–5: 2217.18; 1108.59; 554.29; 277.15; 138.57. F16% 1–5: 2534.40; 1267.20; 633.60; 316.80; 158.40. C: negative control; without drug activity. *p<0.05 statistical difference of F14% compared with experiment control (*C*. *albicans* without treatment). ^#^p<0.05 statistical difference of F16% compared with experiment control (*C*. *albicans* without treatment). The data are expressed as the mean ± SD of three separate experiments.

Based on the fungicidal effect of PRPe on the clinical isolates of *C*. *albicans*, we evaluated whether this extract incorporated in MTS was able to kill the *Candida* isolates. As shown in Fig 3, the fungicidal evaluation of the F14% and F16% formulation, for selected clinical isolates, were able significantly reduce (3log10 CFU/mL) cell viability starting from 1108.59–1267.20 μg/mL of TPC expressed in gallic acid equivalents for F14% and F16%, respectively, compared to control (*p*<0.05). In addition, F16% was more effective than F14% since it inhibited 50% of isolate growth at the above concentration, with a substantial reduction in cell viability (3log10 CFU/mL) at 2534.40 μg/mL of TPC expressed in gallic acid equivalents compared to control (*p*<0.05); this reduction was not concentration-dependent. In comparison, the images (Fig 3) illustrate a qualitative analysis of the inhibition in fungal growth at these concentrations.

### Effect of the mucoadhesive thermoresponsive systems containing propolis in an experimental model of vulvovaginal candidiasis

Preclinical trials are essential for the evaluation of new therapeutic options. In this sense, we applied a robust VVC model using *C*. *albicans* with standard clinical isolates and additionally included a fluconazole-resistant clinical isolate to evaluate the efficiency of the MTS.

**Fig 4:**
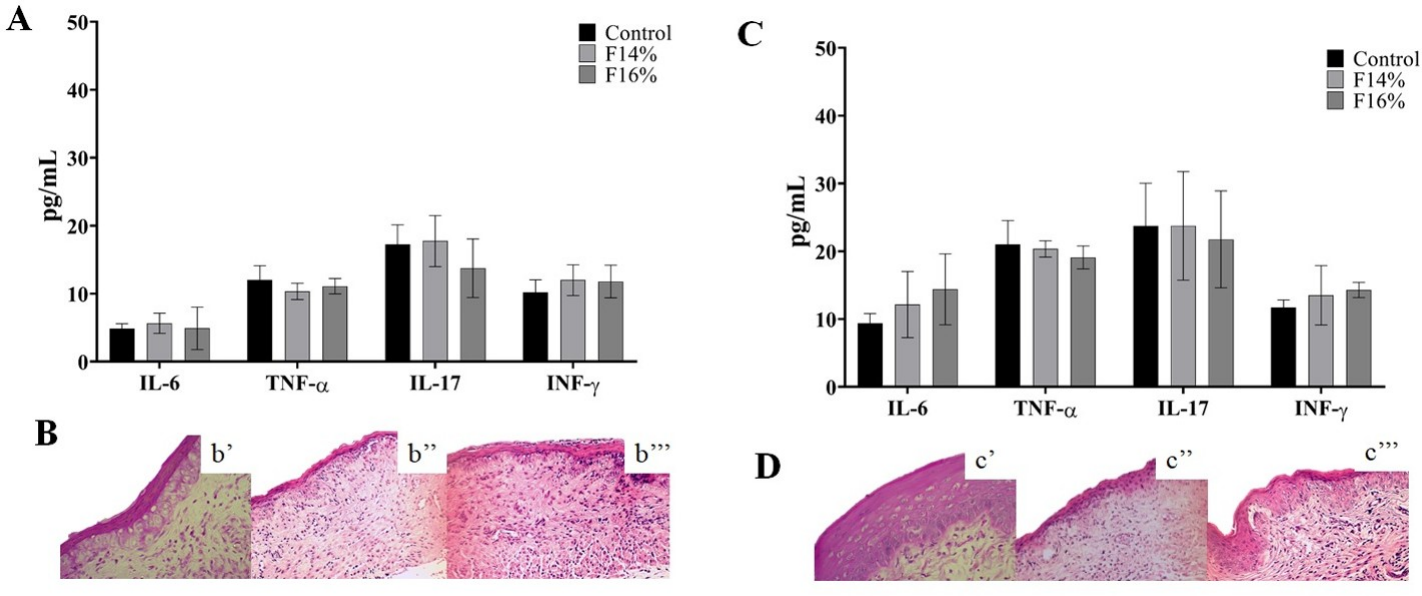
The toxicity assay of mucoadhesive thermoresponsive formulations containing propolis at 14% (F14%) and 16% (F16%) in the vaginal mucosa. Control group: uninfected and untreated mice. F14%: uninfected mice treated with MTS-PRP with propolis at 14%. F16%: uninfected mice treated with MTS-PRP with propolis at 16%. (A) Cytokine dosage of intravaginal lavage of uninfected mice treated for 7 days. (B) Histopathological representation of uninfected mice vaginal mucosa treated for 7 days. b’: control group. b”: F14%-treated group. b”‘: F16%-treated group. (C) Cytokine dosage of intravaginal lavage of uninfected mice treated for 14 days. (D) Histopathological representation of uninfected mice vaginal mucosa treated for 14 days. d’: control group. d”: F14%-treated group. d”‘: F16%-treated group. Cytokines: IL-6—interleukin-6; TNF-α—tumor necrosis factor; IL-17- interleukin-17A; IFN-γ—interferon-γ. Assays performed with at least three animals in each group and in triplicate. There were no statistic difference between control and treated groups.

In agreement with the *in vivo* experiments, the formulations F14% and F16% showed no toxicity to the vaginal tissue. Non-infected animals and mice treated with the F14% and F16% formulations showed a vaginal epithelium without inflammatory characteristics and with keratinization similar to the control group (without infection and treatment), as shown in Fig 4. In addition, the cytokine levels were similar between the treated and untreated groups, and no evidence of inflammation was observed in the histopathological images. These acceptable safety results allowed us to develop the protocol in infected animals.

**Fig 5:**
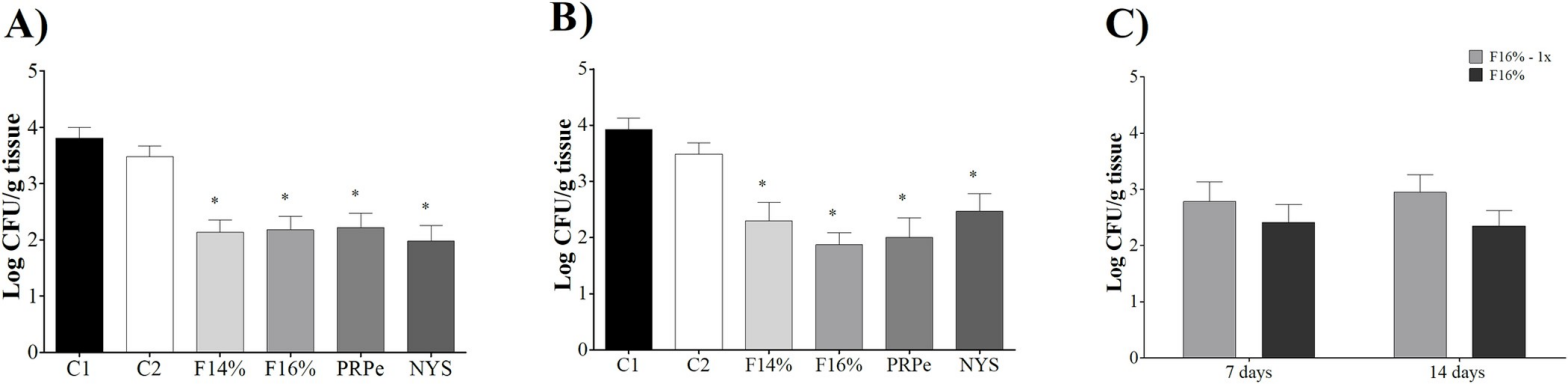
The efficacy of mucoadhesive thermoresponsive formulations with propolis in a *C*. *albicans* experimental vulvovaginal candidiasis. Mice intravaginally infected with 1x106 *C*. *albicans* ATCC 90028. The animals were treated and followed for 7 and 14 days. (A) Fungal burden (Log10 CFU/g tissue) after 7 days of treatment. (B) Fungal burden (Log10 CFU/g tissue) after 14 days (C) Fungal burden (Log10 CFU/g tissue) after 7 and 14 days, treated once and twice a day, with MTS-PRPe at 16%. C1: VVC-infected animals treated with PBS, twice a day. C2: VVC-infected animals treated with mucoadhesive thermoresponsive system without propolis, twice a day. F14%: VVC-infected animals treated with MTS-PRPe at 14%, twice a day. F16%: VVC-infected animals treated with MTS-PRPe at 16%, twice a day. F16%-1x: VVC-infected animals treated with MTS-PRPe at 16%, once a day. PRPe: VVC-infected animals treated with propolis extract, twice a day. NYS: VVC-infected animals treated with nystatin commercial cream, twice a day. The data are expressed as the mean ± SD of two separate experiments. *p<0.05 in relation to control.

The treatment of experimental VVC due to *C*. *albicans* infection was efficient at both evaluated time points, according to Fig 5. The *in vivo* experiments showed a significant reduction in *C*. *albicans* (ATCC) burden (approximately 2log10 CFU/mL) in the PRPe, F14%, and F16%-treated groups, with an NYS-like response, after 7 days of treatment in experimental VVC (Fig 5A) compared to the control group (C1), (*p*<0.05). With an improved response, treatment for 14 days (Fig 5B) further reduced the fungal burden in animals treated with F16%, i.e. greater than 2 log10 CFU/mL compared to the control group (C1) (p<0.05). Moreover, it was observed that F14% was able to maintain antifungal activity until the last analyzed day, unlike nystatin with a small increase. In addition, we tested F16% with once daily dosing. Our data show that treating 1x per day was as efficient as twice per day, with no statistical significance between them (Fig 5C).

There was no statistically significant difference between the control groups (C1 and C2), as C2 animals were treated only with MTF without PRP (*p*<0.05) (Fig 5A and 5B).

**Fig 6:**
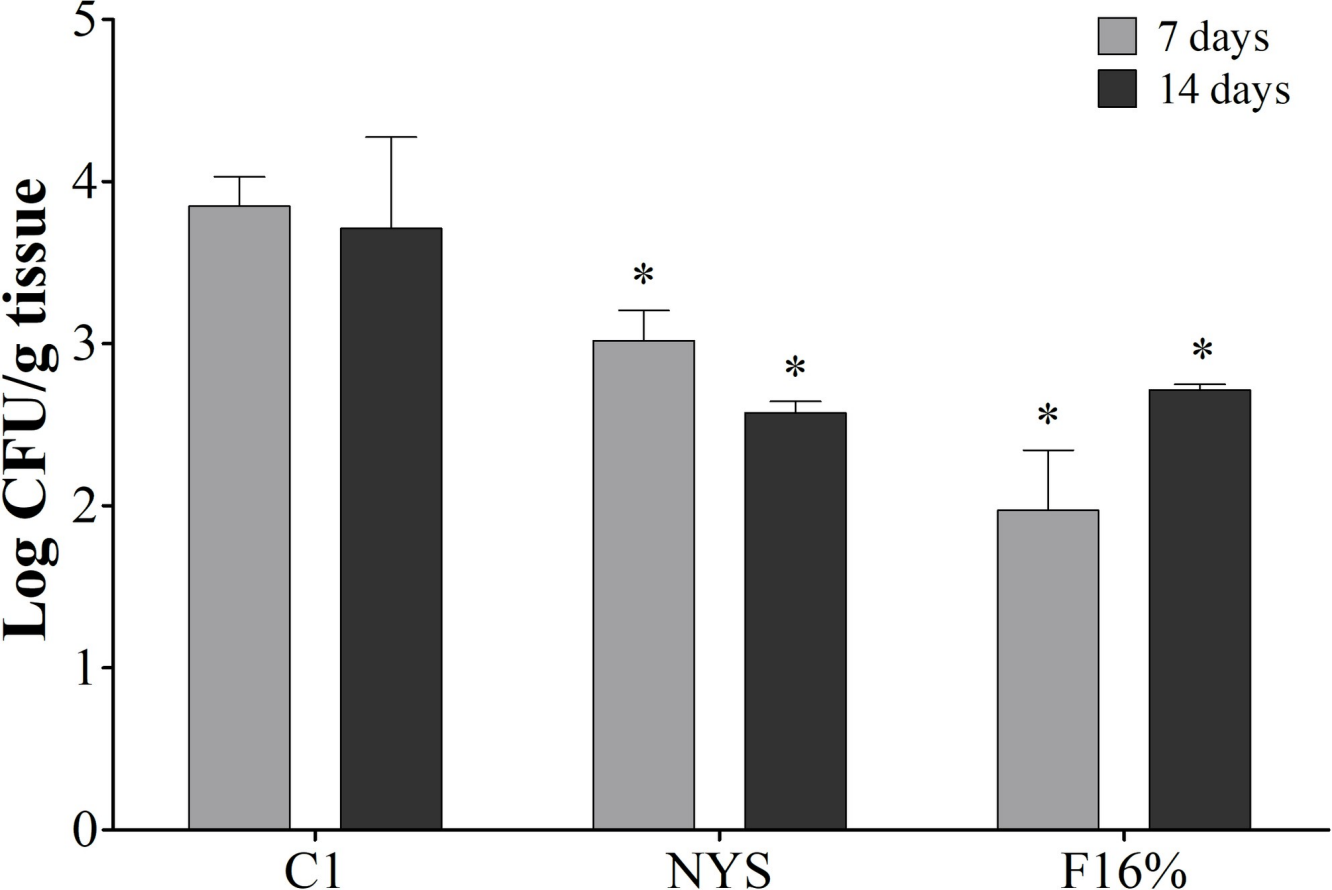
The efficacy of mucoadhesive thermoresponsive formulation with propolis at 16% in a *C*. *albicans* experimental vulvovaginal candidiasis infected with a clinical isolate FCZ-resistant. Mice intravaginally infected with 1x106 *C*. *albicans* clinical isolate fluconazole (FCZ)-resistant. The animals were treated and followed during 7 and 14 days, results were expressed by the fungal burden (Log10 CFU/g tissue). C1: control group; infected animals treated with PBS, twice a day. NYS: infected animals treated with nystatin commercial cream, twice a day. F16%: infected animals treated with MTS-PRPe at 16%, twice a day. *p<0.05 in relation to control.

Based on the good results with F16%, we tested this formulation on clinical isolates of *C*. *albicans*. Curiously, Fig 6 shows that the F16% was also efficient in the treatment of experimental VVC with a fluconazole-resistant clinical isolate, leading to a reduction in fungal burden of approximately 2 log10 CFU/mL, at both analyzed time points. It must be considered that this result is better than that found for NYS. Even with satisfactory *in vitro* results for this antifungal, *in vivo* there was an apparent resistance to killing *C*. *albicans*, especially when compared to treatment with F16%.

Estrogen promotes modifications in the vaginal epithelium that are typical of women childbearing age and allow the infection, adhesion, and survival of the pathogen in the genital tract. S1 Fig in S1 Data illustrates the histological and SEM consequences of pseudoestrus status in the vaginal epithelium, where the organization and keratinization evaluated after 7 days of follow-up (S1B and S1D Fig in S1 Data) were maintained for up to 14 days, the final time point at which our animals were followed and treated, with similar quality (S1C Fig in S1 Data).

**Fig 7:**
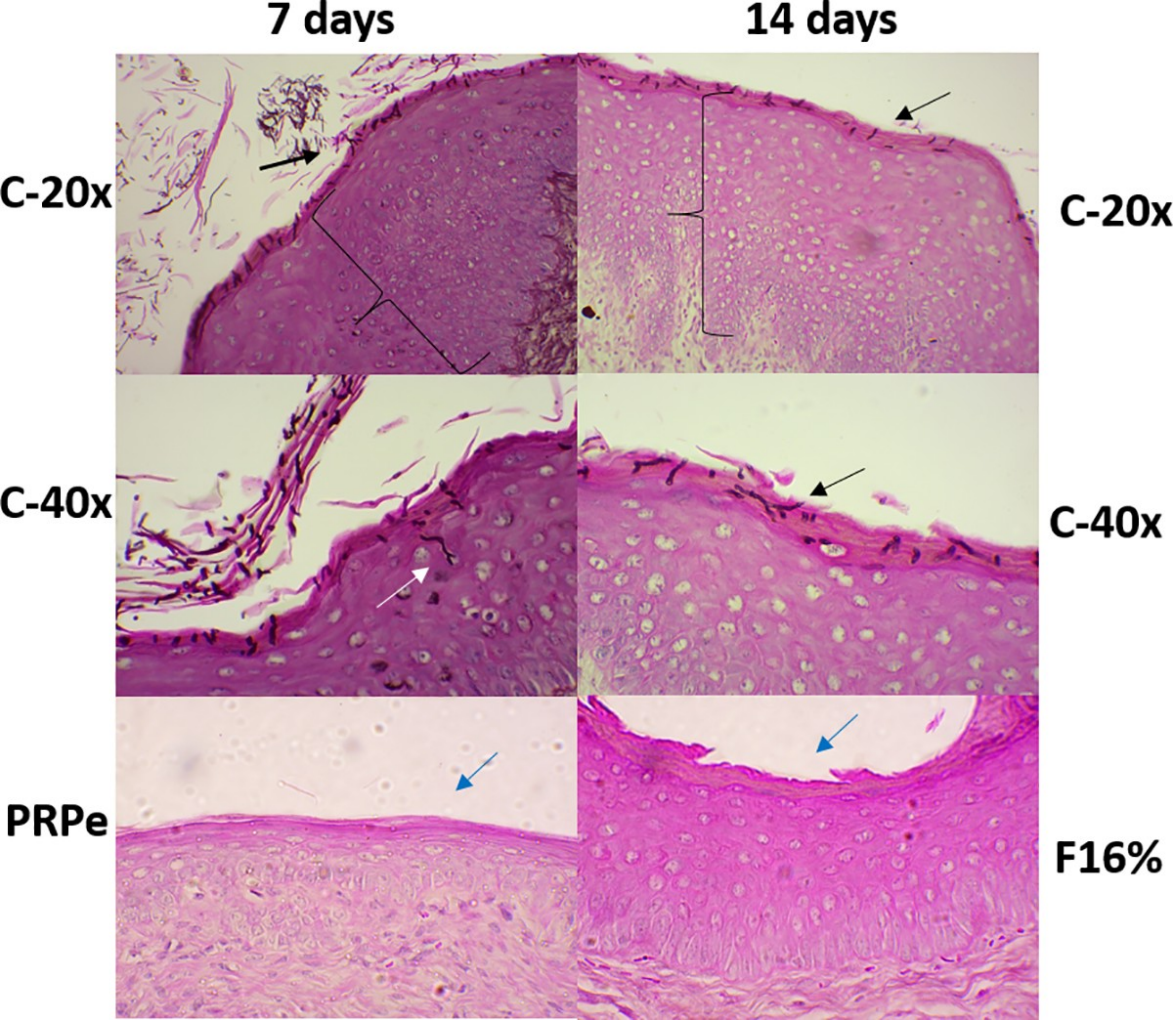
Histopathological examination of the Balb/c mice vaginal mucosa illustrating the effect of propolis extract (PRPe) and mucoadhesive thermoresponsive system containing propolis (MTS-PRPe) treatment in an experimental vulvovaginal candidiasis by *C*. *albicans*. (C-20x) Control group: untreated animal after 7 and 14 days of infection (20x). (C-40x) Control group: untreated animal after 7 days of infection (40x). (PRPe) Group with VVC infection treated with PRPe, twice a day, for 7 days (40x). (F16%) Group with VVC infection treated with MTS-PRPe at 16%, twice a day, for 14 days (40x). Black arrows: presence of *C*. *albicans* hyphaes in the keratinized layer. White arrow: presence of *C*. *albicans* hyphae in the intermediate layer of the vaginal epithelium. Black braces: epithelial thickness and edema. Blue arrows: absence of *C*. *albicans* hyphaes in the keratinized layer. Sections were stained with Grocott-Gomori'smethenamine silver (GMS) and counterstained with haematoxylin and eosin (H&E). Representative images being taken over 50–100 fields of view.

In this context, images from Fig 7 agree with the analysis of the fungal burden, showing in the control group (Fig 7A, 7A’, 7B and 7B’) the presence of multiple hyphae of *C*. *albicans* filamentation in the keratinized layer. Fig 7B shows an image from the control group with penetration into the epithelium 7 days after infection, associated with edema and epithelial disorganization. These epithelial changes are directly related to the presence of pathogens. On the other hand, the reorganization of the epithelium and absence of fungi are evident in the treated groups (Fig 7C and 7C').

**Fig 8:**
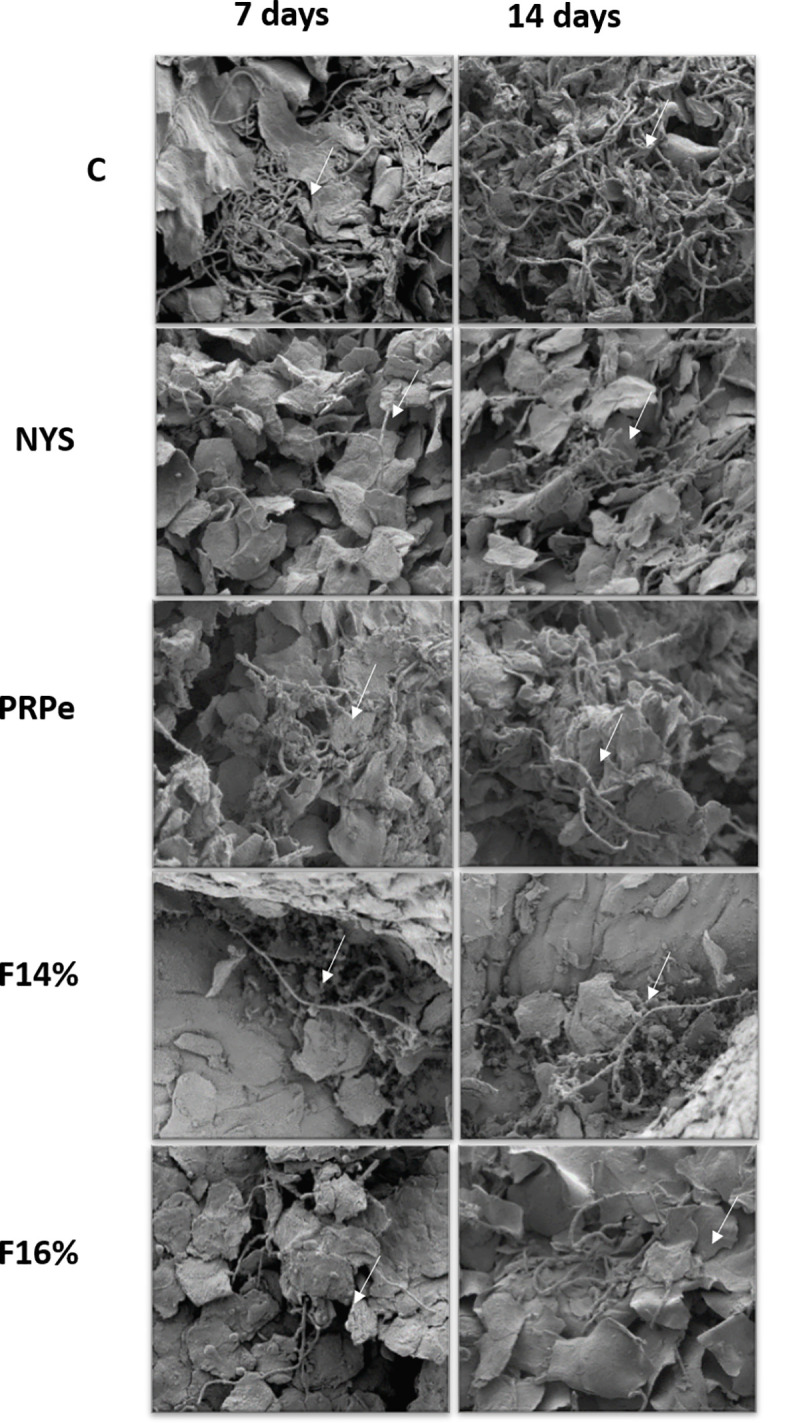
Scanning Electron Microscopy (SEM) images of *C*. *albicans* experimental vulvovaginal candidiasis treated with nystatin (NYS), propolis extract (PRPe), mucoadhesive thermoresponsive system containing propolis at 14% (F14%) and 16% (F16%). (C) Control group: VVC-infected and untreated animal after 7 and 14 days of infection (1200x). (NYS) VVC-infected group treated with NYS, twice a day, for 7 and 14 days (1200x). (PRPe) VVC-infected group treated with PRPe, twice a day, for 7 and 14 days (1200x). (F14%) VVC-infected group treated with F14%, twice a day, for 7 and 14 days (1200x). (F16%) VVC-infected group treated with F16%, twice a day, for 7 and 14 days (1200x). Presence of *C*. *albicans* hyphaes: white arrows. Representative images being taken over 50 fields of view.

Furthermore, Fig 8 strengthens these results as a qualitative analysis of SEM images from vaginal epithelium, showing an expressive reduction in the fungal burden in treated groups compared to control, demonstrating NYS-like behavior.

## Discussion

VVC is one of the most frequent vaginal infections, just behind bacterial vaginosis, and has a direct impact on quality of life in most symptomatic cases. *C*. *albicans* has been reported as the species most commonly involved in genital fungal infections [31]; the first-line treatment includes the use of topical or oral azoles and polyene agents [32]. Nevertheless, VVC treatment has proven to be a challenge in clinical practice, given the predisposing host factors (e.g. hormonal contraceptive and antibiotics use, pregnancy), the limited number of drugs available and the increasing antifungal resistance of pathogens driven by the indiscriminate use of antifungals [33]. In this context, the development of new therapies for VVC treatment should be encouraged in order to achieve greater pharmacological tolerance and low cost options. Thus, this study presented a therapeutic option for the treatment of VVC by means of a preclinical study with better results than the reference drug tested.

The majority of publications on new VVC therapies are based on *in vitro* results. Dalben-Dota et al [24] evaluated the *in vitro* antifungal activity of propolis extract against yeasts *C*. *albicans* and *Candida* non-*C*. *albicans* with a good response compared to nystatin; in the same way, the present study also began with *in vitro* screening and showed that PRPe had excellent antifungal activity on clinical isolates of VVC (Fig 3). Fig 6 shows that PRPe was also effective against a fluconazole-resistant clinical isolate.

We next sought to incorporate PRPe into an available pharmaceutical system for VVC treatment. For this, our group had already invested efforts in the technological development and characterization of an MTS-PRP for various uses [14, 17, 20, 29, 30, 34]. The first step was to evaluate the antifungal activity of the formulations at different concentrations of propolis using *in vitro* assays. The results show that PRPe maintained its antifungal efficacy even when incorporated into the MTS, both for the standard strain and for clinical isolates of VVC (Figs 2 and 3).

Propolis has been studied and explored, since ancient times, in its various biological functions as an antifungal [14, 16, 24, 34, 35], antibacterial [36], anti-inflammatory [37], antineoplastic [38], immunomodulatory [39, 40], tissue repair promoter [41, 42], and antioxidant [43]. These results are attributed to the complex constitution of this extract that acts synergistically, highlighting the phenolic compounds as the main constituents with biological activity. In this context, this compound is attractive for VVC treatment, since it is clinically characterized as an infectious disease associated with an important inflammatory process that results in clinical manifestations (discharge, pruritus, burning, dyspareunia, dysuria) presented by women affected by VVC.

Investing in a pharmaceutical formulation such as MTS-PRPe is very important and useful, since it complements the use of this extract, considering its properties: viscoelastic formulation, resistance to deformation, good retention, thermoresponsivity, prolonged release for more than 24 hours, and easy administration and dispersion in the vaginal mucosa [17, 23].

The systems had their pH set to 7.0 using triethanolamine due to the presence of Carbopol 934P, a polymer with acidic characteristics. This pH value utilized for the best mechanical and rheological characteristics of the systems [14, 17, 21]. Considering that the mouse vagina has a neutral pH, while the environment of the human vagina id acidic, as well as the small amount of formulation administered, the formulation pH cannot change this physiological condition due the gradual swelling of Carbopol chains. Moreover, the neutral pH of the formulations (14% and 16%) may balance the vaginal pH, reducing the marked acidity of VVC.

When the T_sol/gel_ of a vaginal formulation is lower than 20–25°C, a gel can be formed at room temperature, hindering handling and administration. On the other hand, if the T_sol/gel_ is higher than 37°C, the liquid can remain inside the vaginal cavity that can lead to flow and loss of the formulation from the site of action [17]. In this study, the systems displayed a preliminary sol-gel transition temperature near 20°C, indicating that they can be maintained under refrigerated temperatures, like many other medicines. However, the time spent preparing the formulation in the vaginal applicator was enough to increase the temperature, thereby reducing the risk of discomfort. Afterwards, the systems were applied before the sol-gel transition, ensuring a good distribution and contact with the vaginal mucosa.

Knowing the *in vitro* profile of this formulation and considering the low toxicity of this extract [44, 45]; the PRPe cytotoxic concentration was higher than the therapeutic concentration in this study. Thus, the use of this compound was considered safe for the next step of this study, which was to assess the *in vivo* antifungal activity of MTS-PRPe in a pre-clinical model of VVC. At this step, the results were even more promising, since the formulations tested at different concentrations of propolis (F14% and F16%) had expressive antifungal activity at both evaluated time points. Also, we noted that administration of the F16% formulation showed similar results in terms of decreased fungal load when treatment was given once or twice daily (Fig 5C). Finally, the anti-fungal action of the propolis formulations was well illustrated and exemplified in Figs 7 and 8. Moreover, according to Fig 7, a reduced inflammatory response after treatment with propolis could be inferred with the reorganization and tissue recovery of the vaginal mucosa. Therefore, this type of therapy is extremely interesting as it establishes ease of administration for future clinical studies. Moreover, it is important to note that the results obtained with the propolis formulations were similar or even superior to those with nystatin, a commercial reference treatment used as first line in VVC therapy [32].

These results are encouraging since they make possible the application of formulations in the clinical phase, considering the properties of adhesion and persistence of the compound in the vaginal mucosa, as well as the possibility of one application daily, meeting the interests of patients and facilitating adherence to treatment.

## Conclusion

Our results support the use of PRPe as a promising option treatment for vaginal infections caused by *C*. *albicans*, as well as the MTS, especially because of its convenient physicochemical properties which makes its use easier and more comfortable, with better results than nystatin. Therefore, these results motivate us to invest efforts in the next steps of new antifungal agent development through clinical trials.

## Supporting information

S1 Data (DOCX)
