# Supplementary material for: Preclinical approaches in vulvovaginal candidiasis treatment with mucoadhesive thermoresponsive systems containing propolis
Source: PLoS One. 2020 Dec 11;15(12):e0243197. doi: 10.1371/journal.pone.0243197 (PMC7732059; doi:10.1371/journal.pone.0243197)
Supplement: S1 Data — (DOCX) [file pone.0243197.s001.docx]

**S1 Data**

**Preparation and characterization of propolis extract**

**Propolis sample and quality control**

A sample of propolis (PRP) was obtained from an apiary of *Apis mellifera L*. bees located in a native forest with a predominance of *Baccharis dracunculifolia* (*Asteraceae*) and eucalyptus. The classification of this PRP is ‘type BRP’, a typical PRP from the northwest of Parana state, Brazil [1]. The sample was macroscopically analyzed for odor, flavor, and aspect. Afterwards, this sample was subjected to refrigeration for 24 h, crushed in an industrial blender, and stored in a plastic bag at temperature of -18°C until further analysis. The loss upon drying was determined by the gravimetric method, using an analytical moisture analyzer; the results were calculated per 100 g of drug, expressing the average of three determinations [2]. Moreover, 1.0 g of PRP was added 10 mL of petroleum ether and subjected to heating until boiling during 5 min; this process was repeated twice. The ethereal fraction was discarded, and the remaining PRP sample was dried in a desiccator and subsequently submitted to reweighing after cooling in order to determine the wax content [2]. For the determination of the ash content, a PRP sample was incinerated and subjected to calcination in a muffle oven. The sample was placed in a desiccator for cooling and subsequent weight determination. The technique was repeated until a constant weight was achieved [3]. PRPe was evaluated as a decoction of comminuted propolis (1 g) in 100 g of purified water for 10 min. Then, 20 g of the filtrated was exactly weighed and evaporated. The soluble fraction in ethanol was also evaluated by preparing PRPe ethanol (1%, w/w) under reflux [2]. All results are expressed as percentages (%, w/w) representing the mean of three replicates.

**Preparation and characterization of PRP extract solution (PRPe)**

PRPe 30% (w/w) was prepared using ethanol 96°GL by turbo extraction [2]. An amount of 3 g of PRPe, with occasional shaking, was concentrated in a water bath (100°C). The concentrated material was dried on an Ohaus-MB 200 infrared analytical balance (Pine Brook, NJ, USA) at 110°C; the results indicate the dryness residue (DR) value. To estimate the inherent variability of each determination, six replicates were carried out [3]. The pH, relative density and alcohol content were also evaluated according to American and Brazilian Pharmacopeias [3,4]. The total polyphenol content (TPC) of PRPe was determined by the Folin-Ciocalteau method, with some modifications [3,5]. Briefly, 2.0 μL of PRPe were mixed with 10 mL of purified water and 1.0 mL of Folin-Ciocalteau (Galena, Campinas, SP, Brazil), then the volume was completed to 25 mL using an aqueous solution of sodium carbonate 14.06 % (w/v). After 15 min, the absorbance was measured using a spectrophotometer (UV-1650PC, Shimadzu, Tokyo, Japan) at a wavelength λ = 760 nm. A calibration curve of gallic acid was used as a reference [4] and the TPC was expressed as the amount of total polyphenol content (%) in the extract, corresponding to the mean of six determinations.

**Results**

**Characterization of propolis extract (PRPe)**

The results obtained for the physicochemical characterization of PRP and PRPe are summarized in Table S1 and Table S2, respectively. The PRP sample had an aromatic odor and a spicy flavor, adhesive aspect and a greenish-yellow color, confirming the characteristics of PRP collected in the north and northwest of Parana (Brazil).

**Table S1. Physicochemical analysis of the propolis (PRP) sample**

| **Physicochemical analyzes** |  | **Results (mean ± s)** |
| --- | --- | --- |
| **Loss on drying (%, w/w)**  **Wax content (%, w/w)**  **Ash content (%, w/w)**  **Extractive water content (%, w/w)**  **Content soluble in ethanol 96 ° GL (%, w/w)** | | **6.87 ± 0.15**  **41.62 ± 0.31**  **2.53 ± 0.08**  **15.29 ± 0.11**  **54.40 ± 1.74** |

**Table S2. Physicochemical analysis performed for the propolis extractive solution (PRPe) (30%, w/w)**

| **Physicochemical analysis** |  | **Results (mean ± s)** |
| --- | --- | --- |
| **pH**  **Relative density (g/mL)**  **Dryness residue (%, w/w)**  **Alcohol content (%, w/w)**  **Total polyphenols content (%, w/w)** | | **5.32 ± 0.08**  **0.8617 ± 0.0005**  **16.11 ± 0.07**  **68.47 ± 1.25**  **2.68 ± 0.10** |

The quality control shown in Table S1 and S2 highlights important points for the safe use of the PRP sample and PRPe. The loss on drying aims to evaluate the moisture content of the drug, and the value found for PRP (6.87%) indicates that it was collected from the innermost sites of the hive and was well-conditioned [6]. Other important factor is the determination of the extractive content, which is a key parameter in the optimization of the extraction process. It allows for evaluating the ability of water and ethanol to remove substances present in PRP. As PRP is a resinous material, its solubility in water is limited. Thus, the extractive content in water for PRP was 15.29% (w/w). For the extractable fraction in 96° GL ethanol, the value found was 54.40% (w/w). These values are in agreement with the recommended values [2]. PRP contains versatile biological activities, with broad applications in medicine, cosmetics, and the food industry [2,6]. An efficient way to extract PRP is turbo extraction. Polyphenols are one of the major groups of compounds that occur in plants. Among the approximately 350 compounds isolated in PRP, the great majority are polyphenols [7]. The result obtained for the TPC of PRPe was 2.68% (w/w), comparable with the values found in other studies (1-7.28%) [7,8].

**S1 Fig. Histopathological and Scanning electron microscopy (SEM) examination of the vaginal mucosa of Balb/c mice illustrating the pseudoestrus status (PS) weekly performed with 0.1mg of 17 β-estradiol valerato.** A) Uninfected animals without PS (40x). B) Uninfected PS animals after 7 days (40x). C) Uninfected PS animals PS after 14 days (40x). D) SEM of uninfected PS animals after 7 days (1200x).





**References**

1. Marcucci MC, Ferreres F, Garcıa-Viguera C, Bankova VS, De Castro SL, Dantas AP, et al. Phenolic compounds from Brazilian propolis with pharmacological activities. J. Ethnopharmacol. (2001) 74, 105–112. doi: 10.1016/ S0378- 8741(00)00326-3.
2. Bruschi ML, Klein T, Lopes RS, Franco SL, Gremião MPD. Contribuição ao protocolo de controle de qualidade da própolis e de seus extratos. Rev Bras Cienc Farm (2002) 23(2): 289-306.
3. United States Pharmacopeia. Rockville: United States Pharmacopeial Convention (2015).
4. Farmacopeia brasileira. Brasília: Agencia Nacional de Vigilância Sanitária (2010).
5. Teixeira, EW, Negri G, salatino A, Stringheta PC. Seasonal variation, chemical composition and antioxidant activity of Brazilian propolis samples. Evid Based Complement Alternat Med (2010) 7(3):307-15.
6. Bruchi ML, Panzeri H, de Freitas O, Lara RHG, Gremião MPD. Sistemas de liberação de fármaco intrabolsa periodontal. Ver Bras Cienc Farm (2006) 42(1):29/47.
7. Dalben-Dota KF, Faria MG, Bruschi ML, Pelloso SM, Lopes-Consolaro ME, Svidzinski TI. Antifungal activity of propolis extract against yeasts isolated from vaginal exudates. J Altern Complement Med. 2010 Mar;16(3): 285-90. doi:10.1089/acm.2009.0281.
8. Miguel MG, Nunes S, Dandlen SA, Cavaco AM, Antunes MD. Phenols and antioxidant activity of hydro-alcoholic extracts of propolis from Algarve, South of Portugal. Food Chem Toxicol (2010) 48(12):3418-23. doi:10.1016/j.fct.2010.09.014.
